# Outcomes in Frontal Sinus Fracture Repair: A Comparative Analysis Between Plastic Surgery and Otolaryngology (ENT)

**DOI:** 10.3390/cmtr19030030

**Published:** 2026-06-23

**Authors:** Lasya P. Marla, Caroline E. Baker, Macy E. Mitchell, Samuel Girian, John A. Girotto, Anna R. Carlson

**Affiliations:** 1 College of Human Medicine, Michigan State University, Grand Rapids, MI 49503, USA; marlalas@msu.edu (L.P.M.); mitch875@msu.edu (M.E.M.); 2 Pediatric Plastic and Craniofacial Surgery, Helen Devos Children’s Hospital, Grand Rapids, MI 49503, USA; caroline.baker@corewellhealth.org (C.E.B.); john.girotto@helendevoschildrens.org (J.A.G.)

**Keywords:** frontal sinus fracture, plastic surgery, otolaryngology, surgical outcomes, NSQIP

## Abstract

This study conducts a comparative analysis of surgical outcomes in patients who underwent FSF repair by a plastic surgeon versus an ENT using a national database. A retrospective analysis was conducted on patients who underwent surgical treatment of FSFs by a plastic or ENT surgeon using the de-identified American College of Surgeons National Surgical Quality Improvement Program (ACS NSQIP) database. Patients were identified based on surgical CPT codes. Data extracted included primary surgeon specialty and patient demographics, comorbidities, and surgical outcomes. Statistical analysis was performed using Fisher’s Exact test and the Wilcoxon Rank-Sum test, with a *p*-value < 0.05 representing statistical significance. A total of 111 patients were analyzed, of which 85.6% were male. The mean age was 30.0 years [22.0, 48.0]. There were 70 patients (63.1%) treated by an ENT and 41 (36.9%) by a plastic surgeon. The median operative time was 131.0 min for ENT and 115.0 min for plastic surgery (*p* = 0.19). The median length of postoperative stay was 1.0 day for both groups. Postoperative complications included surgical site infection (SSI), wound disruption, and sepsis in five patients (4.5%). There was no statistically significant difference in the rate of complications between patients who underwent surgery with an ENT surgeon versus a plastic surgeon (*p* = 0.16). There were no statistically significant differences in operative time, length of stay, or complications between patients who underwent FSF repair by an ENT or by a plastic surgeon. Surgeon specialty training does not appear to influence intraoperative or postoperative outcomes. Studies with larger sample sizes may demonstrate statistically significant differences in outcomes.

## 1. Introduction

Frontal sinus fractures (FSFs) account for 5–12% of all maxillofacial fractures [1]. These injuries often result from high-impact events such as motor vehicle accidents, assaults, and sports-related trauma [2]. Owing to their anatomical location, FSFs can lead to life-threatening transcranial and/or intracranial complications (such as meningitis or epidural abscess), along with lifelong functional and cosmetic impairments [3]. FSFs are managed by various specialties, including plastic surgery and ENT. Evidence-based treatment protocols have been developed within each specialty, but there is limited literature comparing outcomes across surgical fields [4,5,6]. Existing studies often have small sample sizes or are confined to single institutions, limiting their generalizability.

Although both plastic surgeons and ENTs are qualified to treat FSFs, the Accreditation Council for Graduate Medical Education (ACGME) outlines different training requirements regarding these injuries. Plastic surgery residents receive substantial training in facial fracture repair and complex soft tissue reconstruction through craniofacial training. ENT residents are extensively exposed to sinonasal and skull-based surgeries [7,8]. Moreover, national facial trauma call data reveal that, at level 1 trauma centers, plastic surgeons treat FSFs in 39.6% of cases, while ENTs treat 23.3% of cases [9]. These differences in training objectives and case volumes between plastic surgery and ENT raise the question of the impact, if any, on FSF post-repair outcomes.

To address this current literature gap, we conducted a comparative analysis of surgical outcomes for FSFs between plastic surgery and ENT using the ACS NSQIP database. This study aims to evaluate perioperative outcomes following FSF repair as reported in the ACS NSQIP database, providing a data-driven framework to inform discussions regarding specialty involvement in FSF management. Given the limitations inherent to national surgical databases, the present study is intended to evaluate short-term perioperative outcomes rather than definitive long-term treatment success following FSF repair.

## 2. Materials and Methods

A retrospective cohort study was performed using the ACS NSQIP inpatient database. Patients who underwent FSF repair between 2015 and 2023 were identified using CPT codes 21343 and 21344. Variables collected included patient demographics, comorbidities, perioperative details, and postoperative outcomes within 30 days of operation; the variables extracted within each category are summarized in Table 1. Statistical analysis was completed using Fisher’s Exact test for categorical variables and the Wilcoxon Rank-Sum test for continuous variables, with statistical significance represented by *p* < 0.05.

## 3. Results

A total of 111 patients underwent repair of FSFs during the specified time frame. Patient demographics are presented in Table 2A. The majority of patients identified as White males in their thirties. Preoperative comorbidities are presented in Table 2B. There was no statistically significant difference in comorbidity frequency between the two groups. Over one-third of the cohort were smokers, and medication-treated hypertension was the most common health comorbidity (*n* = 22, 19.8%).

Patients who underwent operative repair of an FSF were more often treated by an ENT physician than a plastic surgeon (*n* = 70 vs. 41, respectively). Perioperative variables between the two specialties are detailed in Table 3. There was no statistically significant difference in operative time (131 min vs. 115 min, *p* = 0.19) or length of hospital stay between the two groups (1.0 for both groups, *p* = 0.90).

The overall complication rate for the cohort was 4.5%. Eight postoperative complications were experienced by five patients in the ENT-treated group, and no complications were experienced in the plastic surgery-treated group, which was not statistically significant (7.1% vs. 0%, *p* = 0.16) (Table 4). The eight postoperative events included deep SSI, superficial incision SSI, wound disruption, pneumonia, pulmonary embolism, and DVT (Table 4). One patient required reoperation. Further analysis did not show significant associations between health comorbidities and the occurrence of postoperative events. Table 5 displays comorbidities with greater than two observations stratified by the groups with and without complications.

## 4. Discussion

Our national study of patients undergoing FSF repair reveals no statistical differences in intraoperative measures or postoperative complications available in the ACS NSQIP database between plastic surgeons and otolaryngologists. Additionally, there were no significant differences in preoperative characteristics between patients treated by either specialty, indicating that both services manage comparable patient populations. Complications occurred infrequently in the cohort, at a rate of 4.5%, and were limited to the ENT-treated group, although there was no statistically significant difference compared to the plastic surgery group.

Both plastic surgeons and ENTs receive extensive training in the management of facial trauma in high-acuity settings during residency and beyond. The type of surgeon assigned to an FSF case may depend on hospital protocol, surgeon availability, institution-specific facial trauma coverage systems, or concurrent injuries [10]. In our analysis, FSFs were more often repaired by an ENT physician than a plastic surgeon, which contrasts with national call data (39.6% for plastic surgery vs. 23.3% for ENT) [9]. Importantly, despite differences in specialty-specific training pathways, comparable perioperative outcomes were observed between groups, suggesting that assigning available surgical teams for FSF management does not appear to compromise immediate patient care. This may reflect variations in institutional coverage patterns, while also highlighting the limited representation of other surgical specialties involved in FSF management within the ACS NSQIP database.

Our data reveal a short hospital stay, low 30-day complication rate, and low reoperation rate for both plastic surgery- and ENT-treated patients, which is consistent with published FSF data for both of the specialties individually [3,4,6,11,12,13]. Our findings add to this body of research by directly comparing the specialties and demonstrating that operative outcomes are not associated with surgeon specialty. These findings may support institutional flexibility in assigning available consulting surgical teams for acute FSF management, particularly in resource-limited hospitals with limited subspecialty facial trauma coverage. However, these findings should not be interpreted as definitive evidence of equivalence between training pathways or long-term reconstructive outcomes.

Our study is not without limitations. NSQIP relies on the fidelity of data from multiple institutions with varying practices in data collection, introducing a potential confounder. Additionally, the database lacks several fracture-specific variables that are highly relevant to FSF management, including posterior table involvement, degree of fracture displacement, nasofrontal outflow tract injury or CSF leak. These factors strongly influence operative decision making, surgical complexity, and postoperative complication risk. The database also does not include cases by other surgeons treating FSFs, such as neurosurgeons and oral maxillofacial surgeons. This restricts the scope of cases, even while utilizing a national dataset. FSF management and outcomes are also dependent on many features of the injury, such as the degree of fracture displacement, involvement of the nasofrontal outflow tract (NFOT), and occurrence of a cerebrospinal fluid leak [12,13,14,15]. The ACS NSQIP database does not capture the aforementioned fracture-specific severity variables, precluding risk adjustment for preoperative presentation of patients between specialties. Fractures vary in etiology, injury pattern, and the functional and aesthetic sequelae. Although all postoperative complications in our study occurred in ENT-treated patients, this did not reach statistical significance, and it is possible that this cohort consisted of more complicated injuries. For example, FSFs involving NFOT have statistically significantly higher complication rates [15]. Without characterizations of the fractures in the national database beyond the anatomic location and the CPT code recorded at the time of operation, the impact of fracture complexity on the results cannot be assessed.

Furthermore, FSFs are associated with several delayed complications, including chronic sinusitis, osteomyelitis, and meningitis, which may develop months to years after the initial injury or repair. Because ACS NSQIP only captures 30-day postoperative outcomes, the database is unable to evaluate many clinically meaningful long-term complications and functional outcomes relevant to FSF management. As a result, our study is limited to assessing short-term perioperative safety rather than comprehensive long-term treatment success.

Our study comprises a relatively small cohort, introducing the risk of type II error, suggesting that a true difference in complication risk between specialties may not have been detected with the available statistical power. Therefore, the absence of statistical significance should be interpreted as hypothesis-generated rather than definitive evidence of equivalence between specialties. Furthermore, the ASC NSQIP only provides 30-day postoperative data, which does not capture long-term functional, aesthetic, or patient-reported outcomes, which are highly relevant in evaluating comprehensive FSF outcomes. Long-term patient outcomes between specialty-specific reconstruction cannot be assessed within this dataset.

These limitations also highlight an important opportunity for future national database development. Expanding ACS NSQIP or similar surgical registries to include fracture-specific injury characteristics, multidisciplinary specialty participation, and long-term craniofacial outcomes could substantially improve the quality of retrospective analyses involving frontal sinus fractures.

## 5. Conclusions

There were no statistically significant differences in operative time, length of stay, or short-term perioperative complications between patients who underwent FSF repair by ENT or plastic surgery. Complications, while rare in this population, were noted only in patients treated by an ENT, although this difference did not reach statistical significance. These findings suggest that immediate perioperative outcomes may not differ substantially based on surgeon specialty within the limits of the ACS NSQIP dataset. Future studies incorporating injury severity, multidisciplinary management patterns, and extended postoperative follow up are needed to better evaluate the impact of surgical specialty on comprehensive FSF outcomes.

## Figures and Tables

**Table 1:** Variables collected.

Category	Variables
Patient Demographics	Sex, age, race, height (in), weight (lbs)
Comorbidities	Smoker within 1 year, dyspnea, diabetes, COPD, hypertension requiring medication
Perioperative	Total operation time (mins), days from admission to operation, days from operation to discharge
Postoperative	Superficial incisional surgical site infection (SSI), deep SSI, wound disruption, pneumonia, pulmonary embolism, deep vein thrombosis, sepsis, return to OR

**Table 2:** (A) Patient demographics; (B) preoperative conditions.

**(A)**			
**Demographic**	**ENT** **(*n* = 70); *n* (%)**	**Plastics** **(*n* = 41); *n* (%)**	***p*-Value**
Sex:			
Female	12 (17.14)	4 (9.76)	0.2849
Male	58 (82.86)	37 (90.24)	
Age (years)	34 (22, 50)	26 (22, 45)	0.1586
Race:			
American Indian/Alaska Native	1 (1.43)	0 (0)	0.6704
Asian	2 (2.86)	1 (3.23)	
Black/African American	6 (8.57)	5 (16.13)	
White	61 (87.14)	25 (80.65)	
Missing		10	
Height (in)	*n* = 69	*n* = 39	0.5520
	68.88 ± 3.20	69.28 ± 3.55	
Weight (lbs)	*n* = 69 176 (155, 202)	*n* = 40 182 (156, 208)	0.4245
**(B)**			
**Condition**	**ENT** **(*n* = 70); *n* (%)**	**Plastics** **(*n* = 41); *n* (%)**	***p*-Value**
Smoker within 1 year	26 (37.14)	15 (36.59)	0.9532
Dyspnea	1 (1.43)	0 (0)	1.0000
Diabetes	6 (8.57)	0 (0)	0.0831
COPD	1 (1.43)	0 (0)	1.0000
Hypertension requiring medication	16 (22.86)	6 (14.63)	0.2942
Disseminated cancer	2 (2.86)	0 (0)	0.5299
Open wound/wound infection	3 (4.29)	5 (12.20)	0.1427
Steroid/immunosuppressive therapy	0 (0)	1 (2.44)	0.3694
Malnourishment	1 (1.43)	0 (0)	1.0000
Bleeding disorder	3 (4.29)	1 (2.44)	1.0000
Preop transfusion	2 (2.86)	0 (0)	0.5299
Sepsis within 48 h to surgery	0 (0)	2 (4.88)	0.1343

**Table 3:** Perioperative variables.

Variable	Whole Sample (*n* = 111); *n* (%)	ENT (*n* = 70); *n* (%)	Plastics (*n* = 41); *n* (%)	*p*-Value
Total operation time (mins)	123.0 (77.0, 177.0)	131.0 (93.0, 181.0)	115.0 (67.0, 165.0)	0.1889
Total hospital LOS (days)	1.0 (0, 1.0)	1.0 (0, 1.0)	1.0 (0, 1.0)	0.6726
Time from admission to operation (days)	0 (0, 0)	0 (0, 0)	0 (0, 0)	0.9030
Time from operation to discharge (days)	1.0 (0, 1.0)	1.0 (0, 1.0)	1.0 (0, 1.0)	0.8707

**Table 4:** Postoperative complication.

Complication	Whole Sample (*n* = 111); *n* (%)	ENT (*n* = 70); *n* (%)	Plastics (*n* = 42); *n* (%)	*p*-Value
Superficial incisional SSI	1 (0.90)	1 (1.43)	0 (0)	1.0000
Organ/space SSI	2 (1.80)	2 (2.86)	0 (0)	0.5299
Wound disruption	1 (0.90)	1 (1.43)	0 (0)	1.0000
Pneumonia	1 (0.90)	1 (1.43)	0 (0)	1.0000
Pulmonary embolism	1 (0.90)	1 (1.43)	0 (0)	1.0000
DVT	1 (0.90)	1 (1.43)	0 (0)	1.0000
Sepsis	1 (0.90)	1 (1.43)	0 (0)	1.0000
Had at least one of the above complications	5 (4.50)	5 (7.14)	0 (0)	0.1556

**Table 5:** Postoperative complications stratified by preoperative comorbidities.

Comorbidity	Complication (*n* = 5); *n* (%)	No Complication (*n* = 106); *n* (%)	*p*-Value
Diabetes	0 (0)	6 (5.66)	1.0000
Inpatient	3 (60.00)	40 (37.74)	0.3737
Smoker within 1 year	3 (60.00)	38 (35.85)	0.3565
Hypertension	2 (40.0)	20 (18.87)	0.2570
Open wound/infection	1 (20.00)	7 (6.60)	0.3170
Bleeding disorder	0 (0)	4 (3.77)	1.0000

## Data Availability

The data that support the findings of this study are available from the American College of Surgeons National Surgical Quality Improvement Program (ACS NSQIP) Participant Use Data File. Restrictions apply to the availability of these data, which were used under license for this study and are not publicly available from the authors. Data may be obtained from the American College of Surgeons in accordance with their data use policies.

## Abbreviations

The following abbreviations are used in this manuscript:

ACS	American College of Surgeons
NSQIP	National Surgical Quality Improvement Program
SSI	Surgical site infection
ACGME	Accreditation Council for Graduate Medical Education
COPD	Chronic obstructive pulmonary disease
CPT	Current Procedural Terminology
DVT	Deep vein thrombosis
ENT	Otolaryngology
FSF	Frontal sinus fracture
IRB	Institutional Review Board
LOS	Length of stay
NSFOT	Nasofrontal outflow tract
OR	Operating room
